# Calcifying Pseudoneoplasm of the Neuraxis, Cerebellum and Cognition: A Rare Opportunity to Learn More

**DOI:** 10.7759/cureus.3982

**Published:** 2019-01-29

**Authors:** Bhaskar Thakur, Simon Riches, Angela Costello, Miren Aizpurua, Istvan Bodi, Keyoumars Ashkan

**Affiliations:** 1 Neurosurgery, King's College Hospital, London, GBR; 2 Psychology, King's College Hospital, London, GBR; 3 Psychiatry, King's College Hospital, London, GBR; 4 Pathology, King's College Hospital, London, GBR

**Keywords:** cognitive functions, posterior fossa, cerebellum, calcifying pseudoneoplasm of the neuroaxis, capnon, neuro-psychiatric rating scales & assessment schedules, neuro-pathology, neuro-oncology, neuro imaging

## Abstract

Calcifying pseudoneoplasms of the neuraxis (CAPNON) are rare tumours. We describe a CAPNON in the posterior fossa and its associated neuropsychological sequelae to provide further evidence for the role of cerebellum in cognitive function. We report the clinical details, imaging, pre-operative neuropsychological assessment, histological features and management of a patient with such a tumour in the posterior fossa location. Detailed pre-operative neuropsychological assessment identified a number of cognitive deficits that had the hallmarks of dysexecutive syndrome. Post-surgery, there was considerable improvement, most notably on processing speed tasks and selected executive tests. This rare case provides further evidence for the role of cerebellum in cognitive function.

## Introduction

Calcifying pseudoneoplasms of the neuraxis (CAPNON) are rare tumours of unknown origin that can occur anywhere within the neuraxis. They are benign lesions that mimic ossified vascular lesions clinically and radiologically [1]. Since they were first identified and described in the late seventies [2], only 45 CAPNON cases have been reported [3]. Of these cases, 33 were intracranial and 12 were spinal. Although it has recently been suggested that these lesions are more aggressive than had been previously thought [3], gross total resection is considered curative and recurrence has been rare among the cited cases and no deaths have been shown to be directly associated with CAPNON [4].

Recent shifts in the study of the cerebellum have shown the centrality that this area of the brain has to cognitive function [5]. We present the fourth case of cerebellar CAPNON and the first case in the literature to document pre/post operative cognitive changes with neuropsychological assessments.

## Case presentation

History and presentation

**Figure 1:**
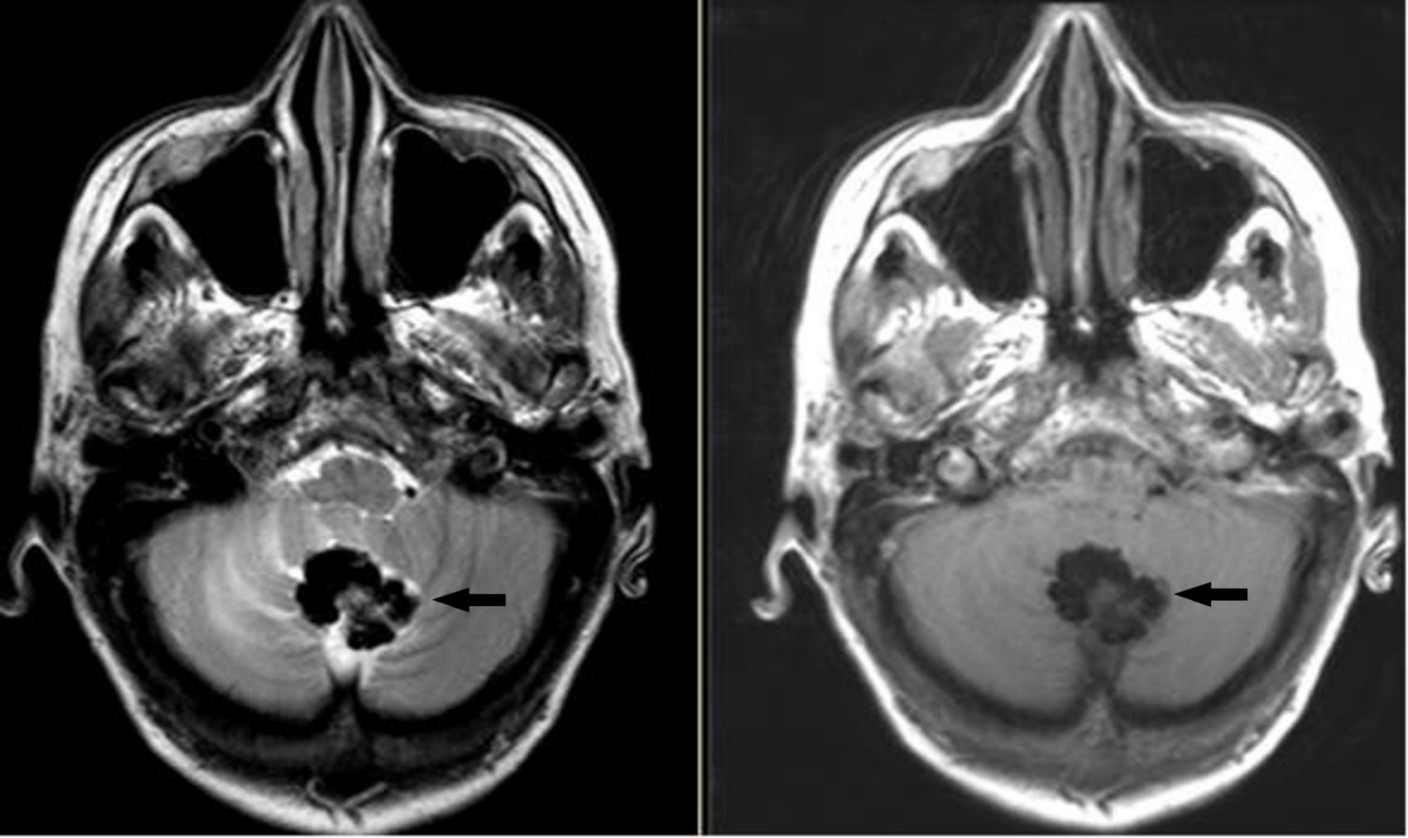
Preoperative MRI T2 (left) and T1 (right) Axial MRI images. Black arrows show the CAPNON. CAPNON: Calcifying pseudoneoplasms of the neuraxis; MRI: Magnetic resonance imaging.

The patient is a 67-year-old female who is ambidextrous but uses her right hand for writing. She presented with a two-year history of difficulty walking. She noticed that her right leg would sometimes drag on the floor. She also thought there had been a decline in her memory and concentration. On examination, the patient appeared to have a late onset cerebellar syndrome and was referred for neuropsychological assessment. Magnetic resonance imaging (MRI) revealed a calcified mass with marked T1 and T2 hypointensity suggestive of a calcified meningioma or oligodendroglioma (Figure 1). Ventriculomegaly was noted on the initial study with no periventricular lucency. There were no features of raised intracranial pressure in the history and no papilloedema on fundoscopy, with a conclusion that there was no active hydrocephalus.

**Figure 2:**
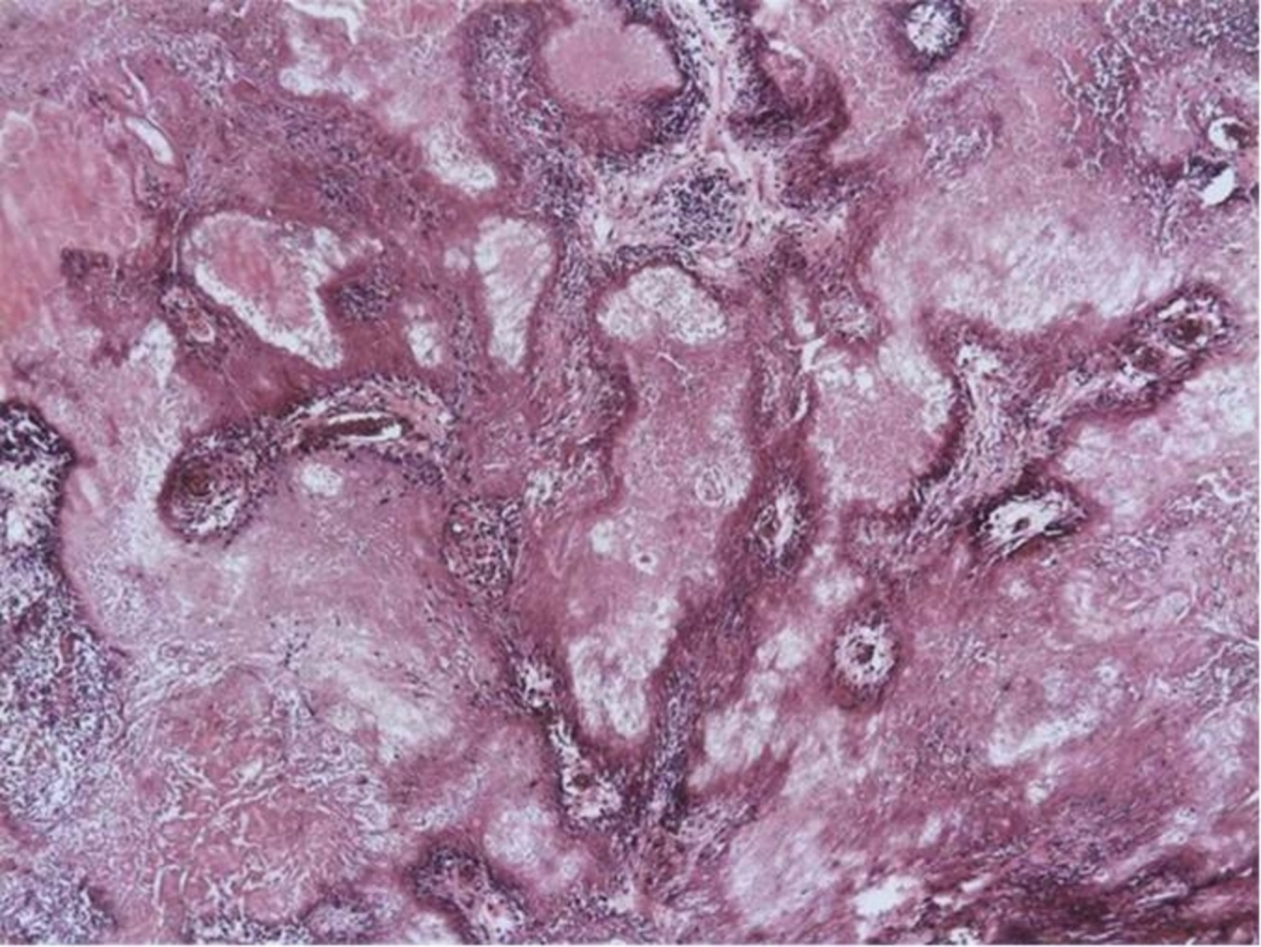
Histology Calcifying pseudoneoplasm of the neuraxis. Coalescing and multilobulated amorphous material with mineral deposition entrapping some reactive brain tissue with mild chronic inflammation (Haematoxylin & eosin, X4 magnification).

The patient underwent a posterior fossa craniectomy for excision of the lesion in November 2013. Histological examination of the decalcified specimen revealed coalescing, multilobulated amorphous material with mineral deposition (Figure 2). Around and among the mineral deposits, there was some atrophic and reactive brain tissue. The deposited material was relatively paucicellular and there was only very mild chronic inflammatory reaction noted around the calcified areas. There were tiny areas with metaplastic bone formation and occasional concentric psammoma body-like structures were also seen. Immunohistochemistry revealed moderate numbers of Cluster of Differentiation 68 (CD68) positive macrophages and prominent astrocytic gliosis by glial fibrillary acidic protein (GFAP) in the brain tissue around the lesion. Epithelial membrane antigen (EMA) and pan-cytokeratin showed no specific immunoreactivity. Ki67 revealed only very occasional proliferating nuclei. Special stains (Grocott, Ziehl-Neelsen, PAS and Gram) were negative for microorganisms. The features were consistent with CAPNON.

There were no post-operative complications and the patient made a good recovery. There was an improvement in coordination and gait following the operation as well as the cognitive improvements detailed below. No cerebrospinal fluid (CSF) diversion was required at any point. An MRI one year post operation has not shown any evidence of recurrence.

Neuropsychological assessment and findings

This was conducted pre-operatively and three months post-operatively, in October 2013 and February 2014 respectively. The same battery of tests was used on each occasion with parallel versions where available. Premorbid level of functioning was assessed pre-operatively with the Test of Premorbid Functioning (TOPF) – UK Version [6]. Intellectual functions were assessed with the ten core subtests of the Wechsler Adult Intelligence Scale IV (WAIS-IV) [7]. Memory functions were assessed with the Recognition Memory Tests for Words and Faces [8], and Brain Injury Rehabilitation Trust (BIRT) Memory and Information Processing Battery (BMIPB) [9]. Verbal recall and list learning language functions were assessed with the McKenna Graded Naming Test [10,11]. Perceptual functions were assessed with the incomplete letters and silhouettes (VOSP) [12]. Executive functions were assessed with the Hayling Sentence Completion Test and the Brixton Spatial Anticipation Test [13], the Delis–Kaplan Executive Function System (D-KEFS) [14], letter fluency category fluency, and Colour-Word Interference subtests.

Intellectual Functions

Pre-operatively the findings were: on the WAIS-IV Full Scale score (FSIQ) = 89, Verbal Comprehension score = 100, Perceptual Reasoning = 88, Working Memory = 95 and Processing Speed = 79 (Table 1). On the TOPF her estimated premorbid FSIQ was 100. Her scores on the Perceptual Reasoning and, more particularly, on the Processing Speed subscales were weaker than her estimated upper average premorbid level of functioning. Post-operatively, on the WAIS-IV FSIQ = 96, Verbal Comprehension = 107, Perceptual Reasoning = 88, Working Memory = 97, Processing Speed = 94. Her scores on the Processing Speed subtests were considerably better post-operatively being in the average range and now in keeping with her estimated premorbid level of functioning.

**Table 1:** Pre- and post-operative test scores 95% confidence intervals 1: Age adjusted scaled scores (range 1-20, mean = 10, SD = 3) 2: Scaled scores (range 1-10, mean = 6) WAIS-IV: Wechsler Adult Intelligence Scale IV; BMIPB: BIRT Memory and Information Processing Battery; VOSP: Visual Object and Space Perception Battery; D-KEFS: Delis–Kaplan Executive Function System.

Instrument	Subtest	Pre-op score	Description	Post-op score	Description
WAIS-IV	Verbal Comprehension	100	50^th^ (94-106)	107	68^th^ %ile (CI 101-112)
	Similarities ^1^	9		10	
	Vocabulary ^1^	10		12	
	Information ^1^	11		12	
	Perceptual Reasoning	88	21^st^ (82-95)	88	21^st^ %ile (CI 82-95)
	Block Design^1^	8		8	
	Matrix Reasoning ^1^	8		8	
	Visual Puzzles^1^	8		8	
	Working Memory	95	37^th^ (89-102)	97	42^nd^ %ile (CI 90-104)
	Digit Span ^1^	9		10	
	Arithmetic ^1^	9		9	
	Processing Speed	79	8^th^ (73-89)	94	34^th^ %ile (CI 86-103)
	Symbol Search^1^	6		10	
	Coding^1^	6		8	
	Full Scale IQ (FSIQ)	89	23^rd^ (85-93)	96	39^th^ %ile (92-100)
Forced Choice Recognition Memory Tests	Words	41/50	25^th^ – 50^th^ %ile	45/50	50^th^ – 75^th^ %ile
	Faces	39/50	@25^th^ %ile	42/50	@50^th^ %ile
BMIPB	Story Recall				
	Immediate	16/60	25^th^	26/60	10^th^ %ile
	Delayed	7/60	5-10^th^	22/60	10^th^ %ile
	List Learning	28/75	Below 2^nd^	32/75	5-10^th^ %ile
Cambridge Cognition McKenna and Warrington	Graded Naming Test	24/30	75-90^th^ %ile	28/30	99^th^ %ile
VOSP	Incomplete Letters	19/20		19/30	
	Silhouettes	22/30		21/30	
Hayling and Brixton	Hayling^2^	5	Moderate average	6	Average
	Brixton^2^	2	Abnormal	5	Moderate average
D-KEFS Verbal fluency	Letter fluency	30	Low average	48	Superior
	Category fluency	32	Average	41	High average
D-KEFS Colour-Word Interference Test	Colour Naming^1^	5	Borderline	8	Low average
	Colour Inhibition^1^	7	Low average	10	Average
	Colour Inhibition/Switching^1^	3	Extremely low	11	Average

Memory Functions

Pre-operatively, recognition memory for both verbal and visual material was satisfactory. On the forced choice Recognition Memory Tests for Words and Faces her scores, 41/50 and 39/50, were between the 25th and 50th percentiles and at the 25th percentile, respectively. Post-operatively, her recognition memory remains satisfactory (Words 45/50; Faces 42/50).

Pre-operatively, aspects of verbal recall memory were however poor. On the immediate recall of the story (BMIPB form 1) her score lay at the 25th percentile and on the delayed recall of the story her score lay between the 5th and 10th percentiles. On the list learning task (BMIPB form 1) her score lay below the 2nd percentile. Post-operatively, aspects of verbal recall memory remain quite poor. On the immediate and delayed recall of the story (BMIPB form 2) her scores lie at the 10th percentile. On the list learning task (BMIPB form 2) her score lies between the 5th and 10th percentiles.

Language Functions and Perceptual Functions

Nominal skills and perceptual functions were entirely satisfactory on both occasions (Table 1).

Executive Functions

Pre-operatively, there was patchy impairment on selected executive tests. Her score on the Hayling test was satisfactory lying in the moderate average category. Fluency for words, both phonemic and category, was satisfactory. However, on the Brixton test her score was in the abnormal category and she scored in the extremely low range on the Inhibition/Switching condition of the D-KEFS Colour-Word Interference Test (Table 1). Post-operatively, her performance on the executive tests is however satisfactory. On the Brixton test she scored in the moderate average category and on the D-KEFS Colour-Word Interference Test her score on the Inhibition/Switching condition was in the average range.

## Discussion

The initial presentation of both motor and executive problems is in line with current research. The cerebellum had previously been known primarily for its contribution to motor function [15]. Petersen et al. in 1989 measured brain function with positron emission tomography (PET) to study the anatomical basis of word processing [16]. When participants generated words, a robust response was observed in the right lateral cerebellum. The response was distinct from the expected motor response present in the anterior lobe of the cerebellum, leading them to the conclusion that a cognitive, rather than a sensory or motor computation being related to this activation [16]. Since then a substantial body of work has furthered our understanding of the cerebellum and role in cognition independent of motor functions [5,17,18]. The cerebellum is interconnected with the contralateral cerebrum primarily through two polysynaptic circuits—an input channel that synapses in the pons and then crosses to the cerebellum and an output channel that projects first to the deep cerebellar nuclei, then to the thalamus, and finally to the cerebral cortex [19]. Schmahmann and Sherman in 1998 described the cerebellar cognitive affective syndrome (CCAS), characterized by disturbances of executive function, impaired spatial cognition, personality change, linguistic difficulties, and found the vermis was consistently involved in patients with pronounced affective presentations [18].

Overall in the case described here, there was considerable improvement in the patient’s processing speed skills. Pre-operatively, she scored in the borderline range on both the symbol search and coding subtests and post-operatively, her scores were in the average and upper low average ranges, respectively. There were notable improvements on both the Brixton and D-KEFS Colour-Word Interference executive tests. The projections to and from the prefrontal cortex to cerebellum would be in keeping with these findings, and further emphasise the cerebellar role in cognitive tasks previously thought to be under the regulation of the frontal lobes [5]. While recognition memory for both verbal and visual material was satisfactory both pre- and post-operatively, aspects of verbal recall memory (list learning) remained quite poor. Language and perceptual functions were entirely satisfactory on both occasions. Overall, our findings suggest a degree of pre-operative frontal lobe dysfunction which was much less apparent post-operatively. The fact that this improvement occurred after removal of the tumour and in the presence of continued ventriculomegaly suggests that these improvements are due to reduced mass effect within the cerebellum, with a resultant improvement in cerebellar function and interaction with pre-frontal cortex [20], as the underlying mechanism.

## Conclusions

This case reports the neuropsychological features of a patient with a calcifying pseudoneoplasm of the neuraxis in the posterior fossa, and subsequent improvement following surgical excision. This provides further evidence for the role of cerebellum in higher function.
